# Comparison of male reproductive success in malaria-refractory and susceptible strains of *Anopheles gambiae*

**DOI:** 10.1186/1475-2875-7-103

**Published:** 2008-06-05

**Authors:** Maarten J Voordouw, Jacob C Koella, Hilary Hurd

**Affiliations:** 1 Department of Biology, University of Victoria, PO Box 3020, Station CSC, Victoria, BC, V8W 3N5, Canada; 2 Division of Biology, Imperial College of London, Silwood Park Campus, Buckhurst Road, Ascot, Berkshire, SL5 7PY, UK; 3 Centre for Applied Entomology and Parasitology, School of Life Sciences, Keele University, Staffordshire, ST5 5BG, UK

## Abstract

**Background:**

In female mosquitoes that transmit malaria, the benefits of being refractory to the *Plasmodium *parasite are balanced by the immunity costs in the absence of infection. Male mosquitoes, however, gain no advantage from being refractory to blood-transmitted parasites, so that any costs associated with an enhanced immune system in the males limit the evolution of female refractoriness and has practical implications for the release of transgenic males.

**Methods:**

Aspects of the male cost of carrying *Plasmodium*-refractory genes were estimated by comparing the males' immune response and reproductive success among strains of *Anopheles gambiae *that had been selected for refractoriness or extreme susceptibility to the rodent malaria parasite, *Plasmodium yoelii nigeriensis*. The refractory males had a stronger melanization response than males from the susceptible line. Four traits were used as correlates of a male's reproductive success: the proportion of females that were inseminated by a fixed number of males in a cage within a fixed time frame, the proportion of females with motile sperm in their spermathecae, the proportion of ovipositing females, and the mean number of eggs per batch.

**Results:**

Although there were significant differences among groups of males in sperm motility and oviposition success, these differences in male reproductive success were not associated with the refractory or susceptible male genotypes. Contrary to expectation, females mated to early emerging refractory males laid significantly more eggs per batch than females mated to later emerging susceptible males. Sperm motility and oviposition success were strongly correlated suggesting that variation in sperm motility influences female oviposition and ultimately male reproductive success.

**Conclusion:**

An increased melanization response in male *A. gambiae *does not diminish male reproductive success under the experimental protocol used in this study. That refractory males induced ovipositing females to lay more eggs than susceptible males is an interesting result for any strategy considering the release of transgenic males. That sperm motility influences female oviposition is also important for the release of transgenic males.

## Background

Parasitic infections exert strong selection on the immune systems of their hosts. However, evolution and maintenance of a host's immune system are thought to be costly via negative effects on other life history traits [1]. Examples of costs incurred by insects include larvae of *Drosophila melanogaster *selected for parasitoid resistance that are less competitive than unselected controls [2] and *Aedes aegypti *mosquitoes selected for early pupation that have a weaker immune response to foreign objects (Sephadex beads) than mosquitoes selected for late pupation [3]. Although immune costs have not always been attributed to precise resistance mechanisms and some mechanisms may not be costly, costs of immune responses that have been identified are a possible explanation for the maintenance of genetic variation in host refractoriness [1,4].

Species of *Plasmodium *that cause malaria in man and other vertebrate hosts impose fitness costs on their mosquito vectors because they reduce fecundity [5-7] and, in some cases, lifespan [8,9]. It is, therefore, expected that anti-*Plasmodium *defense mechanisms have evolved and indeed *Plasmodium *density is reduced by orders of magnitude in many vectors [10-12]. That laboratory colonies of mosquitoes respond readily to artificial selection for refractoriness to *Plasmodium *[13,14] demonstrates that there is genetic variation for this trait. This has been confirmed with recent observations of considerable genetic variation in refractoriness in natural populations [15-17]. Life history costs of refractoriness to *Plasmodium *have been measured in two experiments. *Ae. aegypti *mosquitoes selected for refractoriness to *Plasmodium gallinaceum *had shorter longevity, smaller body sizes and laid fewer eggs than the susceptible population [18]. *Anopheles gambiae *selected for refractoriness to *Plasmodium yoelii nigeriensis *produced fewer offspring when fed on an uninfected blood meal than susceptible mosquitoes after nine generations of selection. However, this difference was observed in only one of the three replicate selection experiments and the effect was not observed after 12 generations of selection [14]. Hence there is some evidence suggesting that in the absence of infection, increased immunity to *Plasmodium *may be costly for female mosquitoes.

While the costs of having refractory genes may be balanced by the benefits in the female, there is no such balancing selection in the male. Male mosquitoes do not blood feed and therefore never encounter malaria parasites. From the perspective of a male mosquito, genes that protect females from *Plasmodium *and all the other parasites (e.g. filarial worms) that come with her blood-feeding habit are not beneficial unless these genes protect males against other pathogens. Genes that have a selective advantage in one sex but not the other are called sexually antagonistic genes and have been demonstrated in *Drosophila *[19]. To date, no one has investigated whether male mosquitoes carrying anti-*Plasmodium *genes experience a fitness cost. Such costs would be important for the release of transgenic mosquitoes carrying anti-*Plasmodium *genes, which will most likely involve releasing males [20].

*Plasmodium*-refractoriness in *A. gambiae *is likely to be associated with more than one resistance mechanism, including parasite lysis [21] and melanization [13,14]. Two independent selection experiments found that refractory *A. gambiae *females evolved a mechanism where they deposit melanin on the surface of *Plasmodium *ookinetes [13,14]. These melanized ookinetes failed to develop to the oocyst stage. More recently it was found that melanization occurs after ookinetes were killed by another mechanism [22]. Regardless of whether it is a primary cytotoxic immune response to *Plasmodium *[23], the melanization response is an attractive candidate to test for sexually antagonistic immune genes. It can be assayed in both male and female mosquitoes by inoculating them with negatively charged CM C-25 Sephadex beads [24]. *Anopheles gambiae *males from a *Plasmodium*-refractory line were shown to have a stronger melanization response towards Sephadex beads than males from a susceptible line [24]. A selection experiment in *Ae. aegypti *found that evolving a stronger melanization response to Sephadex beads is costly with respect to development time [3]. Hence the melanization response is associated with *Plasmodium*-refractoriness in females, can be assayed in males and is costly, but does it influence male reproductive success in *A. gambiae*?

Like most anopheline mosquitoes, *A. gambiae *mates in male-biased swarms that vary in size from twenty to thousands of individuals [25,26]. In the field, females are rapidly mated and leave the swarm following insemination [27]. Polyandry or female multiple mating is therefore rare in the field [< 3%; reviewed in [28]] suggesting that post-copulatory sperm competition is not important [29]. Females store the sperm in their single spermatheca and are capable of laying up to nine batches of eggs in the laboratory with an average of ~100 eggs per batch [30]. In contrast, males return to the swarm after mating [31,32] and laboratory experiments have demonstrated considerable variation in male reproductive success [33,34]. Male reproductive success can be partitioned into its constituent components: insemination success, oviposition success, and hatching success. This partitioning is useful because different mechanisms influence the different components. For example, insemination success (measured as the proportion of females with sperm in their spermatheca) increases with the size of the swarm [35], the number of nights the sexes are kept together [36,37], the male to female sex ratio [36,37], male body size [32,38], and male age [37,39]. Oviposition success (measured as the proportion of ovipositing females) depends upon the transfer of male accessory gland fluids (MAGS) [[40,41]; but see, [42]] and on nervous signals in the female indicating that her spermathecae is filled with sperm [42,43]. In *Anopheles stephensi *and *Ae. aegypti*, hatching success (measured as the proportion of eggs that hatch) decreases over successive batches suggesting either depletion or death of viable sperm in the female's spermatheca [30,44]. Hatching success in *Plasmodium*-refractory lines of *A. gambiae *was lower than that in susceptible lines whereas there was no difference in insemination success between the two lines [14]. This observation suggests that *Plasmodium*-refractory mechanisms may interact in different ways with the different components of male reproductive success.

A range of laboratory mating conditions was recently determined for *A. gambiae *(minimal swarm size of 10 males, 24 hour mating period, 2:1 sex ratio) that enabled the detection of genetic differences in male reproductive success among families of full-sib males [45]. The success of this study motivated the experimental design in the present study. To test for a cost of refractoriness in males, male reproductive success was compared between lines of *A. gambiae *that were refractory or susceptible to the rodent malaria parasite, *P. y. nigeriensis*. Two matched pairs of refractory and susceptible lines of *A. gambiae *were used: Keele black refractory (BR) versus Keele black susceptible (BS) and Keele red refractory (RR) versus Keele red susceptible (RS). These lines had originally been selected from the same outbred Keele strain [14]. It was recently confirmed that BR and RR are still refractory and that BS and RS are still susceptible to *P. y. nigeriensis*, three years after the original selection regime. To test whether artificial selection for *Plasmodium*-refractoriness in females resulted in correlated evolution of the immune response in males, the melanization response was assayed in males from the BR, BS, RR and RS lines. For each of these four lines, male reproductive success was assayed by mating males to females from the Keele population. Male reproductive success was measured in four different ways: the proportion of inseminated females, the proportion of females with motile sperm in their spermathecae 14 days after mating, the proportion of ovipositing females, and the mean number of eggs per batch.

There were significant differences in sperm motility and oviposition success among the 16 groups of males but not between refractory and susceptible males. Females mated to black refractory males laid significantly more eggs per batch than those mated to black susceptible males. In this study, a quarter of all surviving females (57/230) that had taken two blood meals and carried sperm in their spermatheca did not oviposit. These females were almost four times less likely to carry motile sperm in their spermathecae than females that had laid eggs at least once. This suggests that sperm motility influences female oviposition and ultimately male reproductive success.

## Methods

### General methods

*A. gambiae *Keele, refractory (R) and highly susceptible (S) strains of mosquitoes were kept in insectaries maintained at a temperature of 27°C, relative humidity of ~70% and a 12:12 light:dark cycle. Adult mosquitoes were kept in 20 cm cubic mesh cages and were fed *ad libitum *on a solution containing 10% glucose, 0.05% para-aminobenzoic acid (PABA), 0.28% streptomycin/penicillin (Sigma-Aldrich, Poole, UK) and distilled water. Larvae were reared in batches of 50 in small plastic containers (10 × 7 × 5 cm^3^) containing 200 ml of distilled water. Larvae were fed 0.03, 0.04, 0.08, 0.16, 0.32 mg of ground Tetramin™ per individual on days 1, 2, 3, 4, 5 and 0.60 mg every day thereafter. For the male reproductive success assays, adults were mated in 30 cm cubic cages made of wood and plastic mesh (hereafter referred to as mating cages).

### Establishment of 16 sub-lines to separate parental and genetic effects

**Figure 1:**
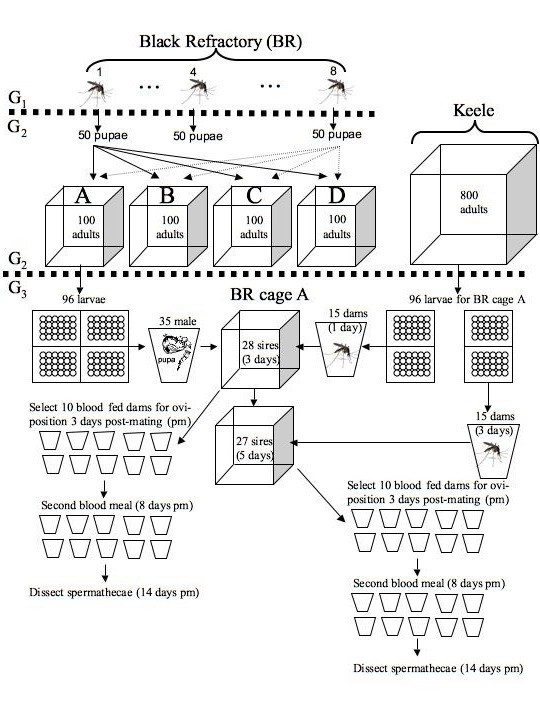
**Experimental design**. For each of the four lines of *Anopheles gambiae *– black refractory (BR), black susceptible (BS), red refractory (RR) and red susceptible (RS) – four sub-lines were established with the same genetic background (only shown for the BR line) by dividing ~400 pupae from 8 first generation (G1) females among four replicate cages (A, B, C and D). The adults in the second generation (G2) produced the males (sires) used in the assay of male reproductive success. The G2 adults from the Keele population produced the females (dams) used in the assay of male reproductive success. All third generation (G3) larvae were reared individually in the wells of 24-well tissue culture plates. In the third generation (G3), a cage of 28 males was obtained for each of the 16 sub-lines (only shown for cage A of the BR line). Each cage of males was mated at 3 and 5 days of age to two different groups of 15 virgin Keele females (aged 1 and 3 days, respectively). Males and females were allowed to mate for 20 hours for each mating day. The females were blood fed two days post-mating (pm). Ten blood fed females were haphazardly selected and allocated to individual oviposition cups 3 days pm. All females were given a second blood meal (8 days pm), were monitored for oviposition (3 – 13 days pm) and were dissected for their spermathecae (14 days pm).

For each of the four lines – black refractory (BR), black susceptible (BS), red refractory (RR) and red susceptible (RS) – four sub-lines were established as follows (Figure 1). Eight batches of 50 larvae were reared for each of the four lines. Each batch of 50 larvae had been obtained from a single *P. y. nigeriensis*-infected female for which the number of oocysts that developed following an infective feed was known [see methods in [14]]. Susceptible females contained hundreds of oocysts whereas refractory females contained none. At pupation, ~50 pupae from each batch were evenly distributed among the four sub-lines for a total of ~100 pupae per sub-line. Hence, within a line, the genetic composition of the four sub-lines was the same. For each of the 16 sub-lines, the pupae were placed in mesh cages (20 cm cubic) and the cages were placed in an alternating sequence (BR, BS, RR, RS, etc) on a single shelf in the insectaria. The purpose of establishing the sub-lines was to separate genetic effects from the parental environment.

### Obtaining refractory and susceptible males and Keele females

For the assay of male reproductive success males from each of the 16 sub-lines were mated to females from the Keele population. To manage the workload, the mothers of the BR and BS males were blood fed one week before the mothers of the RR and RS males (thereby creating a black and red block). Each sub-line was allowed to blood feed for 10 minutes on the forearms of MJV. Three days later, mothers laid eggs in communal oviposition cups and the larvae hatched the following day. For each sub-line, 96 larvae were reared individually in the wells of 24-well tissue culture plates to minimize variation in adult emergence and body size. Larvae were reared on the standard Tetramin™ diet (see General Methods). Due to a dilution error in preparing the Tetramin™ solution, larvae in the black block obtained half the daily food rations on days 5 to 7 as the larvae in the red block. Two days after blood feeding the mothers of the males, the mothers of the females from the Keele population were blood fed. For each block, 768 Keele larvae were reared in the same way as the males.

### Mating assay

For each sub-line, 28 males were mated twice, at 3 and 5 days of age, to different groups of 15 Keele females, aged 1 and 3 days, respectively. The purpose of mating the males to two different groups of females was to determine the repeatability of the mating assay. For each sub-line, the pupae were sexed and ~35 male pupae (range = 31 to 43) were transferred to a separate mating cage over a two-day period. The mating cages (i.e. sub-lines) were scanned every 12 hours to estimate the mean male age at emergence. Two days later the Keele pupae were sexed. For each sub-line, two groups of 15 female pupae were transferred to two separate 700 ml polystyrene cups. All the Keele females emerged on the same night. For each block, the 16 groups of virgin females were randomly assigned to one of the 16 combinations of the 8 mating cages and the 2 mating days. Before adding the females, the number of males in each mating cage was standardized to 28. The first group of females (1 day old) was added to the mating cages at 19:00 and was removed the following day at 15:00. This process was repeated two days later with the second group of females (3 days old). Hence each cage of males had 20 hours to inseminate both groups of females with one day of rest between mating days. All the males were frozen after removing the second group of females. For each of the 16 male cages, the mean wing length was estimated from a sample of 10 males. Wing length was measured as the distance between the allula and the distal fringe using a compound microscope (50× magnification) and an ocular micrometer.

### Oviposition success and sperm motility phenotype

For each sub-line, the two groups of females were kept in separate 700 ml Polystyrene cups. Two nights after the mating assay (i.e. when the first and second group of females were 3 and 5 days old, respectively), the females were blood fed on the arms of MJV for 10 minutes. Females in the red block were starved for 12 hours before taking their first blood meal, but females in the black block were not. The day after the blood meal, ten blood-fed females were haphazardly selected from each cup and were transferred to individual oviposition cups. For the next five days, the oviposition cups were checked every day to record whether the female had laid eggs or not. For each female, the number of laid eggs was counted. Each of the 320 females was given a second opportunity to blood feed five days after their first blood meal. Females were not starved prior to the second blood meal. Each female was fed for 10 minutes on the left or right forearm of MJV and was subsequently transferred to a fresh oviposition cup. After monitoring oviposition for another five days, all females were checked for insemination 14 days after mating. Females were sacrificed by placing them in 70% ethanol for 20 seconds. Females were dissected for their spermathecae in phosphate-buffered solution (PBS). The spermathecae was placed on a slide in 15 μl of PBS and was gently cracked open with fine needles before covering it with an 18 mm cover glass. The spermatheca was immediately checked for the presence of sperm using a light microscope (100× magnification). For inseminated spermathecae, each sperm bundle was observed for 30 seconds to check for the presence of motile sperm. A sperm bundle was defined as motile if at least one motile sperm was observed; it was defined as non-motile if no motile sperm were observed.

### Male melanization phenotype

To test whether refractory males had a more efficient immune response than susceptible males, the melanization phenotype was assayed in male mosquitoes from the BR, BS, RR, and RS lines. For each of the four lines, 200 larvae were reared (see general methods), the pupae were sexed, and the males were blocked by their age of emergence (9, 10 and 11 days after hatching). Each male mosquito was inoculated with one CM C-25 Sephadex bead 24 (± 6) hours after emergence. The beads were hydrated in PBS containing 0.001% methyl green to facilitate visibility. The males were anaesthetized by chilling them on ice for 2 minutes. A single bead and ~0.1 μl of PBS solution was inoculated into the thoracic cavity of the male with a micro-capillary tube pulled into a very fine tip (φ = 40 μm). Inoculated males were placed into 50 ml Falcon tubes that were laid on their sides and that contained moist filter paper and a sugar food source. Males that emerged on days 9, 10 and 11 were assessed for their condition (dead, capable of walking, capable of flying) and then immediately frozen 48, 24 and 12 hours after inoculation, respectively. The following day the thoraxes were dissected in 0.01% methyl green PBS, the bead was searched for up to 10 minutes, and the % melanin cover was estimated for the beads that were found.

### Statistical methods

#### Male reproductive success

There are three binomial measures of male reproductive success: (1) insemination success = the proportion of inseminated females, (2) sperm motility = the proportion of females that had motile sperm in their spermathecae 14 days after mating, and (3) oviposition success = the proportion of females that laid at least one clutch of eggs. There is one normally distributed measure of male reproductive success, the mean number of eggs per batch. Batch refers to the event where a female lays eggs during the five days following a blood meal. The mean number of eggs per batch therefore excludes those events where females did not lay eggs following a blood meal. A generalized linear model (GLM) with a binomial error function is an efficient way to model proportion data. Hence the glm() function in R was used to model the three binomial measures of male reproductive success. For the mean number of eggs per batch, the linear model function in R was used.

#### Mean age of emergence and wing length for males

For the 16 male cages, the mean age at emergence and the mean wing length were normally distributed. These two male traits were modeled as a two-way ANOVA with block (black vs. red), male genotype (refractory vs. susceptible) and their interaction.

#### Covariance in male reproductive success between mating days and effects of male/female age

The 16 cages of males were mated to two different groups of virgin females. Positive covariance between groups of females mated to the same male cage is expected if some cages of males are consistently better at mating than others. For the proportion data, GLM was used to test for male cage effects. For the mean number of eggs per batch, ANOVA was used to test for male cage effects. Separate analyses were conducted for the black and red blocks to ensure that differences between blocks were not causing the male cage effects. The analyses also tested for male/female age effects. Males were mated at 3 and 5 days of age to females, aged 1 and 3 days, respectively. Therefore, the effects of male and female age cannot be separated.

#### Male reproductive success in refractory vs. susceptible genotypes

For each of the four measures of male reproductive success, the data from the two groups of females were aggregated thereby obtaining a single mean for each of the 16 male cages. These means were approximately normally distributed. Male reproductive success was modeled as a two-way ANOVA with block, male genotype, and their interaction. A retrospective power analysis was conducted to determine this study's ability to detect significant differences in oviposition success between the refractory and susceptible males [see Additional file 1].

#### Correlations between the six male traits

The correlations between the six male traits were tested using Pearson's correlation. To control for block effects, the male traits were standardized to z-scores (mean = 0, standard deviation = 1) within each block.

#### Male melanization phenotype

Three-way ANOVA was used to model % melanin cover as a function of line (black vs. red), male genotype (refractory vs. susceptible), and time to melanize a bead (12, 24, 48 hours).

## Results

### Summary of results

Of the 320 females that were assigned to individual oviposition cups, 305 survived to the end of the experiment of which 63% (192/305) laid eggs at least once. Of the 275 females that survived and took two blood meals, females that laid a first clutch were eleven times more likely to lay a second clutch (0.93 = 156/167) than females that did not (0.08 = 9/108; χ^2 ^= 194.28, df = 1, p < 0.0001). Spermathecae were scored for the presence of motile or non-motile sperm for 303 females of which 84% were inseminated (254/303) and 49% (148/303) had at least one motile sperm 14 days after mating.

### Mean age of emergence and wing length for males

The males in the black block emerged 4.1% later than those in the red block (Table 1). The mean wing length of the males in the black block was 6.8% smaller than that of the red block (Table 1). These significant differences in male emergence and male wing length between blocks (Table 2) were most likely caused by the fact that the larvae in the black block received half as much food on days 5 to 7 as those in the red block. The effect of male genotype on the mean age of male emergence depended on block (Table 2). Black refractory males emerged 3.2% earlier than black susceptible males (Table 1) and this difference was statistically significant. Red refractory males emerged 2.1% later than the red susceptible males (Table 1), but this difference was not significant. There was no significant effect of male genotype or the male genotype*block interaction on mean male wing length (Table 2).

**Table 1:** Mean male traits for the refractory and susceptible genotypes

	Black males			Red males		
Trait	Mean	s.e.	n	Mean	s.e.	n
emerge	9.70	0.067	8	9.32	0.058	8
wing	2.61	0.014	8	2.80	0.011	8
p.insem	0.86	0.031	8	0.82	0.037	8
p.motile	0.40	0.059	8	0.58	0.056	8
p.ovip	0.48	0.066	8	0.78	0.063	8
eggs/batch	88.73	3.509	8	101.42	1.067	8
Black block						
	Refractory males			Susceptible males		
Trait	Mean	s.e.	n	Mean	s.e.	n

emerge	9.54	0.034	4	9.86	0.055	4
Wing	2.63	0.022	4	2.60	0.015	4
p.insem	0.82	0.046	4	0.91	0.033	4
p.motile	0.42	0.099	4	0.39	0.078	4
p.ovip	0.46	0.068	4	0.51	0.125	4
eggs/batch	95.86	1.605	4	81.60	4.583	4
Red block						
	Refractory males			Susceptible males		
Trait	Mean	s.e.	n	Mean	s.e.	n

emerge	9.41	0.060	4	9.22	0.078	4
wing	2.80	0.021	4	2.79	0.008	4
p.insem	0.84	0.037	4	0.79	0.068	4
p.motile	0.56	0.059	4	0.59	0.104	4
p.ovip	0.78	0.090	4	0.79	0.103	4
eggs/batch	101.32	2.034	4	101.52	1.081	4

The six male traits are mean time to emerge (emerge), mean wing length (wing), the proportions of inseminated (p.insem), motile sperm bearing (p.motile), and ovipositing females (p.ovip), and the mean number of eggs per batch (eggs/batch). Shown are the means, standard errors (s.e.), and the numbers of male cages (n). For each male cage, emerge is based on ~33 males (range = 24 to 43), wing is based on ~10 males, the proportions (p.insem, p.motile, p.ovip) are based on ~10 females, and eggs/batch is based on ~22 batches (range = 9 to 35).

**Table 2:** Male reproductive success in refractory versus susceptible genotypes

Mean male emergence time (days)					
Effect	df	SS	MS	F	p
block	1	0.582	0.582	42.12	< 0.001
genotype	1	0.016	0.016	1.18	0.299
block:genotype	1	0.256	0.256	18.51	0.001
Error	12	0.166	0.014		
Mean male wing length (mm)					
Effect	df	SS	MS	F	p

block	1	0.139	0.139	115.23	< 0.001
genotype	1	0.002	0.002	1.47	0.249
block:genotype	1	0.000	0.000	0.21	0.652
Error	12	0.014	0.001		
Proportion of inseminated females					
Effect	df	SS	MS	F	p

block	1	0.008	0.008	0.87	0.370
genotype	1	0.001	0.001	0.13	0.721
block:genotype	1	0.021	0.021	2.25	0.160
Error	12	0.111	0.009		
Proportion of females with motile sperm in their spermathecae					
Effect	df	SS	MS	F	p

block	1	0.117	0.117	3.86	0.073
genotype	1	0.000	0.000	0.00	0.959
block:genotype	1	0.003	0.003	0.10	0.752
Error	12	0.363	0.030		
Proportion of ovipositing females					
Effect	df	SS	MS	F	p

block	1	0.363	0.363	9.37	0.010
genotype	1	0.004	0.004	0.09	0.764
block:genotype	1	0.001	0.001	0.03	0.856
Error	12	0.465	0.039		
Mean number of eggs per batch					
Effect	df	SS	MS	F	p

block	1	644.395	644.395	22.31	< 0.001
genotype	1	197.584	197.584	6.84	0.023
block:genotype	1	209.044	209.044	7.24	0.020
Error	12	346.602	28.883		

The six male traits are mean time to emerge, mean wing length, the proportions of inseminated, motile sperm bearing, and ovipositing females, and the mean number of eggs per batch. Each male trait is modeled as a two-way ANOVA of block (black versus red), male genotype (refractory versus susceptible) and their interaction. Shown are the degrees of freedom (df), sum of squares (SS), mean squares (MS), the F-statistic (F), and the p-value.

### Covariance in male reproductive success between mating days and effects of male/female age

**Figure 2:**
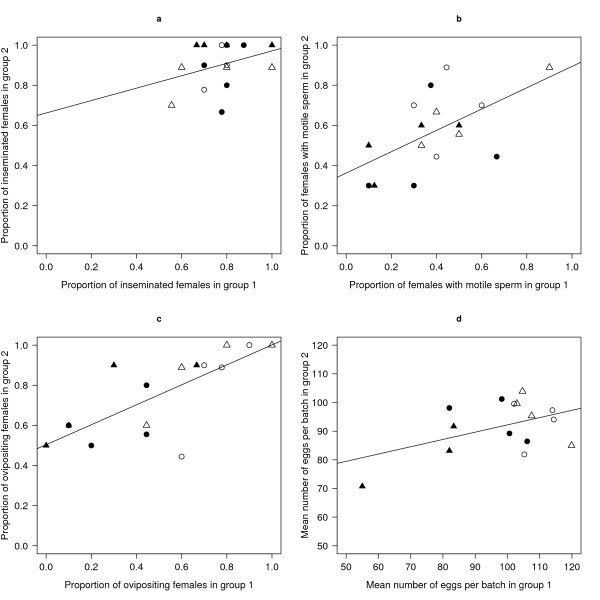
**The correlation in male reproductive success between two days of mating in *Anopheles gambiae***. The correlation between the two groups of females, aged 1 and 3 days, mated to the same cage of males at 3 and 5 days, respectively for four male fitness traits (a) the proportion of inseminated females (r = 0.33, df = 14, p = 0.206), (b) the proportion of females with motile sperm in their spermathecae 14 days after mating (r = 0.58, df = 14, p = 0.019), (c) the proportion of females that oviposited at least once (r = 0.74, df = 14, p = 0.001), and (d) the mean number of eggs per batch (r = 0.47, df = 13, p = 0.078). Females that were mated to BR, BS, RR and RS males are represented by filled circles, filled triangles, open circles and open triangles, respectively. Shown are the lines of best fit from the linear regressions.

The proportion of inseminated females did not differ among male cages in either the black or the red block (Table 3; Figure 2). In both the black and red block, there was a significant effect of male cage on sperm motility and on oviposition success (Table 3; Figure 2). There was no significant effect of male cage on the mean number of eggs per batch (Figure 2) in either the black (F6, 6 = 3.40, p = 0.081) or red block (F7, 7 = 0.39, p = 0.88). The three binomial measures of male reproductive success increased with male/female age: insemination success (0.77 vs. 0.90; Table 3), sperm motility (0.40 vs. 0.57; Table 3), oviposition success (0.50 vs. 0.75; Table 3), but the mean number  of eggs per batch (99 vs. 92 eggs; F1,14 = 2.29, p = 0.066) did not.

**Table 3:** Covariance in male reproductive success between mating days

		Black block			Red block		
id	model	df	dev	AIC	df	dev	AIC
1	p.insem~cage+age	7	9.80	51.22	7	6.56	54.77
2	p.insem~cage	8	15.38	54.80	8	11.00	57.21
3	p.insem~age	14	20.07	**47.49**	14	16.96	**51.17**
4	p.insem~1	15	25.87	51.29	15	20.94	53.15
							
5	p.motile~cage+age	7	7.06	**64.87**	7	4.03	**62.80**
6	p.motile~cage	8	11.85	67.66	8	9.80	66.57
7	p.motile~age	14	22.70	66.51	14	19.73	64.50
8	p.motile~1	15	27.30	69.11	15	24.80	67.57
							
9	p.ovip~cage+age	7	6.40	**60.86**	7	5.50	**52.15**
10	p.ovip~cage	8	34.30	86.76	8	9.01	53.66
11	p.ovip~age	14	26.19	66.65	14	34.39	67.04
12	p.ovip~1	15	52.68	91.13	15	37.11	67.76
							
effect	comparison	Δ df	Δ dev	p	Δ df	Δ dev	p

age	1 vs. 2	1	5.58	**0.018**	1	4.44	**0.035**
cage	1 vs. 3	7	10.27	0.174	7	10.40	0.167
							
age	5 vs. 6	1	4.79	**0.029**	1	5.77	**0.016**
cage	5 vs. 7	7	15.64	0.029	7	15.70	0.028
							
age	9 vs. 10	1	27.90	**< 0.001**	1	3.50	0.061
cage	9 vs. 11	7	19.79	**0.006**	7	28.89	**< 0.001**

For each block (black, red), GLMs were used to model the proportions of inseminated (p.insem), motile sperm bearing (p.motile), and ovipositing females (p.ovip) as a function of male cage (cage) and male/female age (age). For each model the residual degrees of freedom (df), the residual deviance (dev), and Akaike's information criterion (AIC) are shown. For each block, the statistical significances of the age and cage effects were evaluated using log likelihood ratio comparisons of nested models. For each comparison the change in the residual degrees of freedom (Δ df), the change in the residual deviance (Δ dev), and the p-value are shown (p).

### Male reproductive success in refractory vs. susceptible genotypes

**Figure 3:**
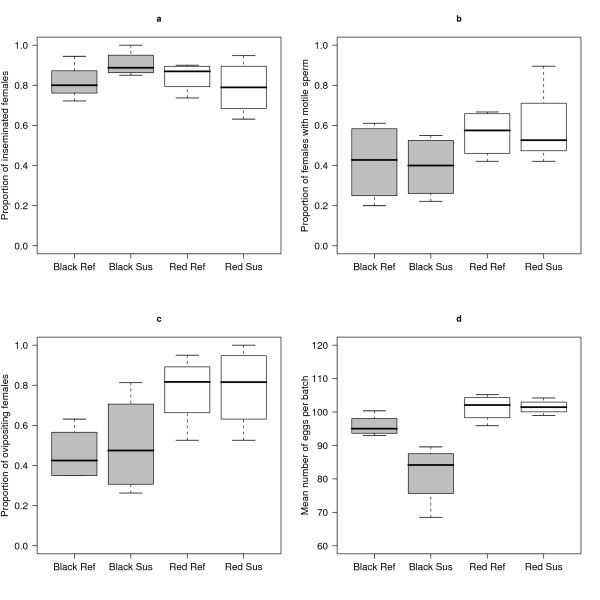
**Reproductive success of *Anopheles gambiae *males from *Plasmodium*-refractory and *Plasmodium*-susceptible lines**. The effect of male genotype (*Plasmodium*-refractory vs. *Plasmodium*-susceptible) and block (black vs. red) on (a) the proportion of inseminated females, (b) the proportion of females with motile sperm in their spermathecae 14 days after mating, (c) the proportion of females that oviposited at least once, and (d) the mean number of eggs per batch. Shown are the medians (bold line), the 25th and 75th percentile (edges of the box) and the minimum and maximum values (whiskers). Each box plot is based on 4 cages.

There was no difference between refractory and susceptible males in insemination, sperm motility or oviposition success (Table 2; Figure 3). There was no effect of block on insemination success or sperm motility (Table 2). Oviposition success in the red block was 1.6 times higher than that in the black block (Table 1). This significant difference in oviposition between blocks (Table 2) was most likely caused by the fact that females mated to red males were starved for 12 hours prior to their first blood meal whereas females mated to black males were not. The red females were therefore more motivated to blood feed than the black females. The results did not change when the mean male age of emergence and mean male wing length were included as covariates.

Females mated to red males laid 14.3% more eggs per batch than females mated to black males (Table 1; Figure 3). This significant difference in eggs per batch between blocks (Table 2) was most likely caused by the differences in blood feeding mentioned above. The effect of male genotype on the mean number of eggs laid per batch depended on block (Table 2). In the black block, the mean number of eggs per batch of the females mated to the refractory males was 17.5% higher than that of the susceptible males (Table 1; Figure 3) and this difference was statistically significant. In the red block there was no difference in the mean number of eggs per batch between females mated to refractory and susceptible males (Table 1; Figure 3). When the mean male age of emergence was included as a covariate, the male genotype*block interaction term and the main effect of male genotype were no longer significant.

### Correlations between the six male traits

After correcting for block effects, insemination success was not correlated with either sperm motility or oviposition success (Table 4). In contrast, sperm motility and oviposition success were significantly correlated (Table 4). There was also a significant correlation between mean male wing length and the mean number of eggs per batch (Table 4).

**Table 4:** The correlation matrix for the six male traits

Male trait	emerge	wing	p.insem	p.motile	p.ovip	eggs/batch
emerge	***	-0.06	0.48	0.07	0.06	-0.18
wing	0.821	***	0.21	0.40	0.47	**0.53**
p.insem	0.062	0.436	***	0.31	0.48	0.11
p.motile	0.797	0.121	0.247	***	**0.61**	-0.01
p.ovip	0.812	0.068	0.057	**0.012**	***	0.35
eggs/batch	0.510	**0.035**	0.687	0.965	0.188	***

Block effects were removed by standardizing the male traits to z-scores within each block. The six male traits include the mean age of emergence (emerge), the mean wing length (wing), the proportion of inseminated females (p.insem), the proportion of females with motile sperm in their spermathecae 14 days after mating (p.motile), the proportion of ovipositing females (p.ovip), and the mean number of eggs per batch (eggs/batch). The correlations are calculated across the two blocks (n = 16 male cages). The Pearson's correlation coefficient and its p-value are shown above and below the diagonal, respectively. Significant correlations and p-values are shown in bold.

### Male melanization phenotype

**Figure 4:**
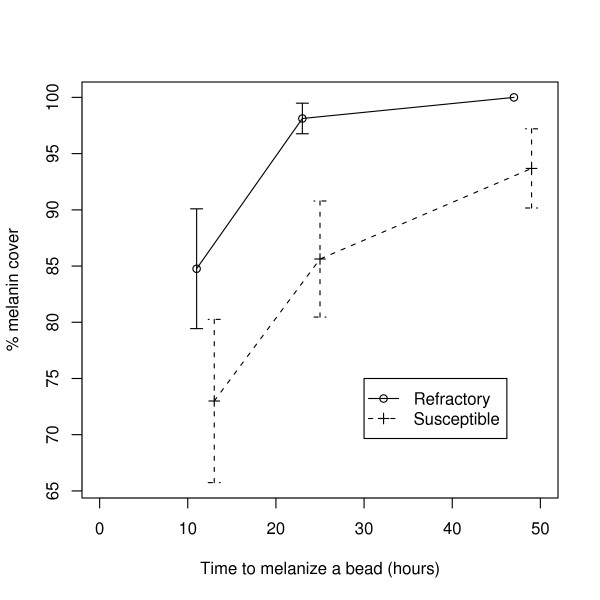
**The effect of *Plasmodium*-susceptibility and refractoriness on melanization response of *Anopheles gambiae *males**. The % melanin cover of a bead increases with the time since the bead was inoculated and the melanization response is more efficient for refractory than susceptible males. Shown are the means and standard errors.

For 116 inoculated males, beads were recovered from 109 individuals, of which 81 were able to fly. Across the black and red lines and the three melanization times, the mean % melanin cover in the refractory males (94 ± 2.3%, n = 54) was significantly higher than that in the susceptible males (84 ± 3.4%, n = 55, F_1,105 _= 6.70, p = 0.011; Figure 4). There was also a significant effect of melanization time; males given 12, 24 and 48 hours to melanize a bead covered 79 ± 4.5% (n = 41), 92 ± 2.9% (n = 32), and 97 ± 1.9% (n = 36) of the bead, respectively (F2, 105 = 12.73, p < 0.001; Figure 4), but there was no difference between the red and black blocks (F_1,105 _= 0.05, p = 0.82). Excluding males unable to fly following inoculation did not affect the results.

## Discussion

No support was found for the main hypothesis of this study, namely that males from populations that are refractory to *Plasmodium *parasites bear constitutive immunity costs and therefore have lower reproductive success than males from susceptible populations. There was no difference between refractory and susceptible males in insemination success, sperm motility, and oviposition success (Table 2, Figure 3). In the black block, contrary to expectation, females mated to refractory males laid significantly more eggs per batch than females mated to susceptible males (Figure 3). This effect of male genotype on the mean number of eggs per batch could not be separated from the earlier emergence of the black refractory males (Table 1). However, the mean male age of emergence was not correlated with the mean number of eggs per batch across the 16 male cages (Table 4). This observation suggests that the difference in the mean number of eggs per batch between the refractory and susceptible males in the black block was due to their genotype rather than to differences in the mean male age of emergence, which were small (3.2%; Table 1). Regardless of causation, females mated to early emerging refractory males laid significantly more eggs than females mated to later emerging susceptible males.

One critique of this study is that the refractory and susceptible males were not put in direct competition with each other. Such an experiment would ideally require the use of genetic markers that are currently not available for these refractory and susceptible strains. Hence the possibility that refractory costs occur when the two male genotypes compete for a common pool of females cannot be excluded. One of the strengths of this study is that independent replicate cages were established for the generation prior to the male fitness assay. Hence parental effects [recently demonstrated in A. stephensi by [46]] were not confounded with the male genotype. Another strength was that, by allowing the males to mate on two separate days, it was shown that this study was sensitive enough to detect significant differences in reproductive success among males. Because the number of males and females in each cage and the amount of time that the sexes were allowed to mate were controlled, these differences in reproductive success among males probably reflect variation in the larval environment, genetics and parental effects. When this variance among males was included into the power analysis [see Additional file 1], it was found that the experiment's power to detect small differences in the proportion of ovipositing females between refractory and susceptible males was low and that replication would have to be quadrupled to be able to detect moderate differences. Previous studies in *A. gambiae *[14] and *Ae. aegypti *[18] detected some female fecundity costs of being refractory to *Plasmodium*, suggesting an explanation for the maintenance of genetic variation for this trait in the field. The present study suggests that refractory genes are neutral with respect to male fitness in a laboratory setting and that males are unlikely to affect the evolution of *Plasmodium*-refractoriness in female mosquitoes. However, biotic factors and environmental stresses encountered in the field could affect male fitness in ways that might alter these conclusions.

Although a cost of refractoriness in males was not detected, refractory males had a more efficient melanization response than susceptible ones. Paskewitz and Riehle [24] also showed that the refractory strain of Collins *et al *[13] had a much stronger melanization response to C-25 Sephadex beads than the susceptible one and that the difference between strains was more pronounced for males than females. Using a time course experiment they showed that the % melanization cover stabilized after 24 hours, which was not the case for our males (Figure 4). In this experiment, variation in the age of emergence (9, 10, 11 days) was confounded with the time given to melanize a bead (48, 24 and 12 hours, respectively), hence the possibility that developmental rates influenced the melanization response, as shown in *Ae. aegypti *[3], cannot be excluded. The important conclusion, however, is that selection of *Plasmodium*-refractoriness or -susceptibility in female mosquitoes resulted in a correlated response in the male immune system.

Most studies on anopheline male reproductive success measure insemination success (i.e. the proportion of inseminated females) and assume that this trait is a good estimate of male reproductive success [34,37,39]. This study emphasizes the importance of partitioning male reproductive success into its constituent components such as insemination success, oviposition success, and the number of eggs per batch [e.g. [45]]. Female oviposition is generally conditional on the female having been inseminated. However, the present study shows that there is some independence between these two components. Twenty-five percent of inseminated, blood-fed females in this study did not oviposit. Males differed in their sperm motility (Figure 2b) and oviposition success (Figure 2c) despite having similar insemination success (Figure 2a). Sperm motility and female oviposition were significantly correlated (Table 4). These two observations suggest that the quality or quantity of the male ejaculate influenced female oviposition. Using the G3 strain of *A. gambia*, it was previously shown that males vary genetically in their ability to induce females to oviposit [45]. The present study suggests a mechanistic link between sperm motility and oviposition.

At this point, the factors that influence sperm motility are not clear. Sperm motility decreases with the time taken to dissect a spermatheca (Voordouw, unpublished data). It appears to be robust to the duration of the observation interval and little additional information is gained from observing the sperm bundle for more than 30 seconds (Voordouw, unpublished data). It is possible that other methodological factors such as the composition of the PBS affect sperm motility [47]. However such methodological factors cannot account for the correlation between sperm motility and female oviposition (Table 4). Perhaps the sperm motility phenotype measured in this study captures some measure of sperm quality such as sperm viability or sperm mobility that have been shown to influence male reproductive success in other systems [48]. Alternatively, sperm motility may represent some measure of sperm quantity if the probability of detecting at least one motile depends on the amount of sperm initially transferred. In the fly, *Scathophaga stercoraria*, the viability of sperm in the female's spermathecae decreases rapidly after mating and was shown to vary significantly among males [49]. In *S. stercoraria*, males have heritable variation in sperm length [50] leading Bernasconi *et al *[49] to speculate that sperm morphology influences variation in sperm longevity. Klowden and Chambers [51] recently reported that *A. gambiae *produces polymorphic sperm and that the female reproductive tract was more likely to contain longer sperm. Long sperm bias in the female spermatheca has also been observed in *Drosophila pseudoobscura *where males produce equal amounts of short and long sperm but only the latter fertilizes the eggs [52]. Future research will focus on how sperm length and sperm motility influence male reproductive success in *A. gambiae*.

A previous laboratory study has shown that large *A. gambiae *males are more likely to acquire mates than small males [38]. In *Anopheles freeborni*, examination of the accessory glands and testes in field-captured males, suggests that large individuals are more likely to mate than small ones [32]. The mechanism that gives larger anopheline males a mating advantage is not known. Large males may be better at displacing small males from their mates (if such take-overs occur in anopheline mating swarms) or may be better at catching and subduing females. Alternatively, large males may have bigger energy budgets that allow them to swarm for longer periods of time as documented in swarming chironomid midges [53]. In this study, male wing length was not correlated with male insemination or male oviposition success (Table 4). However, male wing length was significantly correlated with the mean number of eggs per batch (Table 4); females mated to larger males laid more eggs per batch. A previous laboratory study has shown that *A. gambiae *males prefer to mate with large females that produce larger batches of eggs [35]. Size assortative mating may explain why the mean number of eggs per batch was correlated with male wing length while oviposition success was not.

The three binomial measures of male reproductive success increased over the two days of mating. On the first day of mating, the males were 3-days old-virgins and the females were 1-day-old virgins. On the second day of mating, the males were 5-days-old with one day of mating experience and the females were 3-days-old virgins. Previous work on other strains of *A. gambiae *found that the proportion of inseminated females peaks at seven days of age for both males and females [37], whereas in another study, the proportion of ovipositing females was not affected by the age of the female but was higher for 2-days-old males than 6-days-old males [54]. This study cannot separate male and female age effects or male experience, but this does not influence the comparison of the refractory and susceptible lines as the two mating days were combined.

As mentioned previously, there were significant differences among male cages in oviposition success. These results are similar to a study on the G3 strain of *A. gambiae *that found significant differences among families of full-sib males in oviposition success [45]. In both studies, there was considerable variation in male reproductive success. In the black block of this study for example, oviposition success of the most successful swarm was three times higher than that of the least successful swarm (81% vs. 26%). In the red block, there was a two-fold difference between the most and least successful swarm (100% vs. 53%). If male oviposition success contains a genetic component [e.g. [45]] there is considerable scope for the evolution of male reproductive success. Given plans for the eventual release of transgenic male mosquitoes, identifying and manipulating the genetic mechanisms underlying this variation in male reproductive success may considerably improve their performance in the field.

## Conclusion

In conclusion, *Plasmodium*-refractory males had a more efficient immune response than their susceptible counterparts, but this did not translate into immune-related fitness costs in the laboratory when the immune system was un-challenged. Black refractory males induced ovipositing females to lay more eggs per batch than black susceptible males. Hence the present study suggests that males do not restrict the evolution of *Plasmodium *refractoriness in female mosquitoes in the absence of agents that infect males. Male reproductive success was repeatable across two days of mating with respect to oviposition success and sperm motility. These two measures of male fitness were strongly correlated suggesting a mechanistic link. Future research will be directed towards characterizing what male factors influence sperm motility and oviposition success and whether these can be manipulated to improve the reproductive success of transgenic males in the field.

## Authors' contributions

MJV conceived the idea, ran the experiment and analyzed the data. MJV, JCK and HH designed the experiment, interpreted the data and wrote the paper.

## Supplementary Material

Additional file 1 Power analysis. Post-hoc power analysis to determine the level of replication necessary to detect significant differences in oviposition success between refractory and susceptible males. Click here for file
