# Supplementary material for: Comparison of male reproductive success in malaria-refractory and susceptible strains of Anopheles gambiae
Source: Malar J. 2008 Jun 5;7:103. doi: 10.1186/1475-2875-7-103 (PMC2438369; doi:10.1186/1475-2875-7-103)
Supplement: Additional file 1 — Power analysis. Post-hoc power analysis to determine the level of replication necessary to detect significant differences in oviposition success between refractory and susceptible males. [file 1475-2875-7-103-S1.doc]

**Power analysis**

**Methods**

A retrospective power analysis was conducted for the proportion of ovipositing dams (p). This analysis incorporated the significant among sire cage variance component detected in this study and the binomial variance inherent in proportion data. For each of the 16 cages in our experiment, p was transformed to a normal deviate (x) using the logit link function, x = ln (p/(1 - p)). Across the 16 cages, the mean of x (μ_x_) was 0.778 and the standard deviation of x (σ_x_) was 1.061. The difference in x between refractory and susceptible males (δ) was expressed as a fraction of μ_x_ and the expected means for refractory (μ_ref_) and susceptible (μ_sus_) males were μ_x_-δ/2 and μ_x_+δ/2, respectively (e.g. for δ = 0.8μ_x_; μ_ref_ = 0.467 and μ_sus_ = 1.090). The rnorm() function in R was used to generate ‘n’ random normal deviates (y) for each of the refractory and susceptible male lines using the appropriate mean (μ_ref_ versus μ_sus_) and our estimate of the among cage standard deviation (σ_x_). These deviates were then back-transformed to the probability that a female will oviposit (p_e_) using p_e_ = exp(y)/(1 + exp(y)). The rbinom() function in R was used to generate ‘n’ observed proportions of ovipositing females (p_o_) using the p_e_ probabilities and 20 females per cage thereby incorporating binomial variance into the data. A one-way ANOVA on the p_o_ data was used to test for a significant difference between refractory and susceptible males. For the power analysis the following parameters were varied: σ_x_ (2 levels: 0.010 and 1.061), the number of cages (n) per refractory or susceptible line (3 levels: 8, 16, or 32) and δ (10 levels: 0.2μ_x_, 0.4μ_x_, …, 2.0μ_x_). For each of these 60 combinations of σ_x_, n and δ, 1000 randomizations were run and power was the proportion of ANOVAs where the p-value was statistically significant (p-value < 0.05).

**Results**

The power analysis shows that the experiment (n = 8 replicate cages per refractory or susceptible line) had >80% chance of detecting a statistically significant effect of male genotype on the proportion of ovipositing females when (1) the difference (0.17) between the susceptible (0.76) and refractory lines (0.60) represents 26% of the proportion of females that oviposited (192/305 = 0.63), and (2) the proportion of ovipositing females is influenced only by the genotype of the male (i.e. random differences among cages are negligible; σ_x_ = 0.01; top-left panel in Figure 1). When the significant among-cages variance component (σ_x_ = 1.06) as estimated in this study is incorporated, the power of the experiment (n = 8 replicate cages) to detect statistically significant differences for even the largest values of δ is <70% (bottom-left panel in Figure 1). Given this reality, the sample size would have to be quadrupled (n = 32) to have a 70% chance of detecting the previously mentioned and reasonably small difference of 0.17 between the susceptible (0.76) and refractory (0.60) lines (bottom-right panel in Figure 1).


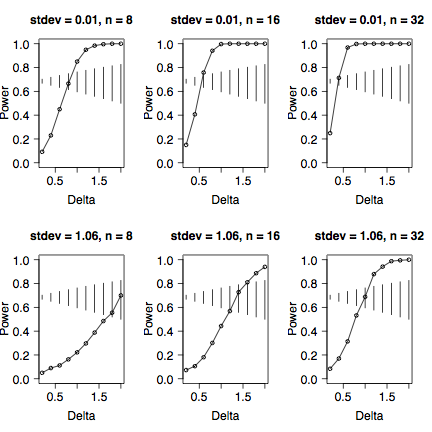


### Figure 1 – Power to detect effect of *Plasmodium*-susceptibility and refractoriness on male reproductive success in *Anopheles gambiae*

The power of our experiment to detect a statistically significant effect of male genotype (refractory versus susceptible) on the proportion of ovipositing females depends on the among-cages standard deviation (stdev = σ_x_), the number of cages (n) per refractory or susceptible line (8, 16, 32), and the difference between the refractory and susceptible males (δ = Delta). In the panels in the top row, random differences among cages are neglible (σ_x_ = 0.01) whereas the panels in the bottom row incorporate the significant among-cages variance component observed in this experiment (σ_x_ = 1.06). Here Delta is expressed as a fraction of the mean (μ_x_) of the normally distributed variable x that is related to the probability that a female will oviposit via the logit link function (see text). In each panel, the top and bottom of the vertical lines show the expected mean proportion of ovipositing females mated to refractory and susceptible males, respectively.
